# Organizational Disaster Communication Ecology: Examining Interagency Coordination on Social Media During the Onset of the COVID-19 Pandemic

**DOI:** 10.1177/0002764221992823

**Published:** 2021-02-05

**Authors:** Wenlin Liu, Weiai (Wayne) Xu, Burton John

**Affiliations:** 1 University of Houston, Houston, TX, USA; 2 University of Massachusetts–Amherst, Amherst, MA, USA; 3 University of Colorado, Boulder, CO, USA

**Keywords:** disaster communication ecology, interorganizational relationships, multiagency coordination, social media

## Abstract

Interagency coordination is crucial for effective multiagency disaster management. Viewing government and emergency management organizations as vital components of citizens’ disaster communication ecology, this study examines how a group of Texas-based public health departments and emergency management offices engaged in interagency coordination during different phases of the COVID-19 pandemic. By analyzing coronavirus-related agency tweets between early February and the end of August 2020, the study assesses two types of interagency coordination: (1) content-level coordination in the form of semantic similarity among the selected public agencies serving different jurisdictions and (2) relational-level coordination in terms of referencing common stakeholders through retweeting coronavirus-related information. Using a granular, four-stage construct of a crisis, results identify stage-based variation with regard to peer-to-peer and federal-to-local coordination. We conclude with theoretical and practical implications for communication ecology and disaster management.

## Introduction

The arrival of the novel coronavirus (known as COVID-19) in early 2020 has been deemed an unprecedented event in modern history (The World Bank, 2020). Several states have struggled to manage this threat within the United States, going from marked lockdowns of their economies to reopenings, to returns, to lockdowns. Texas’s management of this crisis well exemplifies this dynamic: the state went into a phased reopening as early as April 1, 2020, then a complete reopening by May 1; then, because of spikes in COVID-19 cases, the state announced a temporary pause in reopenings on June 25 (Limon, 2020). Such gyrations in the nature of the crisis prompted various emergency management and public health actors at the local, state, and federal levels to adjust their communication to the public and other agencies. The current study examines how these agencies used Twitter to coordinate social media content and interorganizational relationship building across what we propose as four vital, granular stages of a crisis: dormant, latent, active, and plateau. As such, this work offers applied understandings regarding the depth and breadth of such coordination. Additionally, this study’s use of a four-stage, granular construct offers important theoretical and practical implications regarding interagency disaster communication using social media.

## Literature Review

### Disaster Communication Ecology and the Missing Meso-Level Components

Communication ecology is defined as “a network of communication resource relations constructed by individuals in pursuit of a goal and in context of their communication environment” (Ball-Rokeach et al., 2012, p. 4, in Broad et al., 2013). The communication ecology concept posits that individuals may actively construct their information and communication networks from the surrounding environment, consisting of mediated, interpersonal, and organizational connections (Ball-Rokeach et al., 2001; Kim & Ball-Rokeach, 2006). By tapping into such a communication network, individuals can draw the needed resources to make sense of uncertain situations, organize collective action, and achieve goals of various kinds.

Spialek and Houston (2018) define the specific type of communication ecology for disaster coping as “networks of communication resources (e.g., organizations, media, and residents) that are utilized to cope with mental, behavioral, and physical health challenges occurring at different disaster phases” (p. 937). Such ecologies include both micro- and meso-level resources. At the microlevel, interpersonal connections, such as peer citizens or community members affected by the disaster, provide information or other resources for disaster coping and recovery; the meso-level disaster communication resources include information from news media, local emergency management, and other community-based organizations (Spialek et al., 2019).

With the growing research on disaster communication ecology, however, two research gaps remain. First, while crisis and disaster communication literature consistently identify the important role of meso-level institutional actors in providing timely, credible disaster information (e.g., Collins & Kapucu, 2008), organizational communication from emergency management agencies receives disproportionately less attention than interpersonal or mediated communication within the disaster communication ecology research tradition (e.g., Spialek et al., 2019). This discrepancy may be attributed to the low penetration rate (e.g., the limited scope of reach, low level of utilization) of official disaster communication in hard-to-reach communities, such as culturally and linguistically isolated ethnic communities (Peguero, 2006). In these communities, interpersonal communication and ethnic media are more likely to be the trusted or preferred source for disaster information (Liu, 2020).

Second, beyond examining disaster communication from individual agencies, even less research focuses on interorganizational disaster communication—that is, the communication directed at and received from peer disaster management agencies. Disaster management literature has recognized the critical role of “boundary spanners,” the type of organizations that can promote the flow of information exchange beyond the established networks and “act as conveners between various sectors” (St. John & Yusuf, 2019, p. 154). Empirical work suggests that boundary-spanning activities, such as intrasector and cross-sector networking among emergency management organizations, can help exchange timely information and enhance the adaptive capacity of the overall disaster response system (Anthony et al., 2014).

Recognizing the existing gaps, below we focus on examining organizational- and interorganizational disaster communication in the context of social media–mediated communication.

### Government Use of Social Media for Interagency Coordination

Interagency coordination has been widely studied in organizational behavior, public administration, and disaster management. Although various definitions are proposed, we adopt Malone and Crowston’s (1994) classic view of coordination as the management of dependencies between entities, characterized as “the additional information processing performed when multiple, connected actors pursue goals when a single actor pursuing the same goals would not perform” (p. 112). To situate coordination in the specific context of disaster management and communication, extant research has emphasized the post hoc nature of such a process. That is, rather than relying on a preexisting network of partners, disaster response often involves establishing new interorganizational connections with entities such as autonomous relief agencies, government organizations serving at different levels of jurisdictions (e.g., federal vs. local level), media, and private sector organizations (Bharosa et al., 2010). Thus, the post hoc nature makes disaster coordination “a problem of contingency” (Prizzia, 2008, p. 82), where existing organizational structures may be ill-prepared to adapt to the rapidly unfolding situations in a disaster.

Over the past few years, public agencies have been increasingly using social media for disaster (e.g., Liu & Xu, 2019). As citizens also increasingly turn to social media platforms for real-time disaster information (Jin et al., 2014), there are greater public expectations for disaster management agencies to maintain an active social media presence in order to provide timely disaster updates, debunk misinformation, and promote the public’s accountability perceptions toward government agencies during emergencies (Neely & Collins, 2018). Recent research in this area also indicates that public agencies’ social media use has evolved and matured in the sense that more strategic planning and human capital have increasingly gone into some agencies’ disaster communication on social media. Liu and Ni (2020) summarize three ways in which public agencies use social media in the context of natural disaster preparedness, response, and recovery crisis communication: (1) information provision and instruction, which refers to a wide range of activities such as broadcasting disaster-related updates, debunking misinformation, responding to public inquiries, and connecting the public to relevant information resources; (2) community building, the use of social media to produce narratives that help boost community morale and cultivate a sense of togetherness; and (3) interagency coordination and networking. The third category notably leverages social media’s connective function, such as the retweet or mention features on Twitter, to coordinate with and mobilize action from other key players such as peer disaster management agencies, nonprofit and civil society organizations, businesses, or even individual citizens. This interagency coordination function is of particular interest from a communication ecology perspective.

### Social Media–Mediated Coordination

While most disaster management literature examines interagency coordination as joint action facilitated by off-line interorganizational ties (e.g., Bharosa et al., 2010; Malone & Crowston, 1994), we propose two interrelated forms of interagency coordination on social media: (1) the content-level coordination as indicated by the level of overlap or similarity of social media content from multiple disaster management agencies and (2) the relational-level coordination as indicated by the level of overlap or similarity of information sources disaster management agencies seek to promote.

#### Content-Level Coordination

Risk communication literature has long considered the importance of providing consistent information, especially when the risk topic is novel and complex (Renn & Levine, 1991). For example, the World Health Organization’s (2020) guideline to improve risk communication and community readiness to COVID-19 recommends that agencies coordinate “message preparation, consistency, and dissemination” (p. 3).

The coordination of social media content among multiple disaster management agencies can facilitate risk reduction in the following ways. First, receiving consistent messages from multiple official sources can help citizens make sense of equivocal situations and boost trust toward official agencies, both of which are crucial in forming accurate risk perception and promoting individuals’ adherence to the recommended behaviors (Sellnow et al., 2009). Second, with rumors and misinformation disrupting most organization-public disaster communication, communicating consistent information is also instrumental in dispelling misinformation (van der Meer & Jin, 2020).

In the current study, we assess content-level coordination by examining the extent to which an organization’s social media content overlaps with other organizations in topics and themes. Specifically, we adopt the concept of “cultural betweenness” by Bail (2006, p. 11824), which refers to the extent to which an organization’s messages serve as the “bridge” to connect discursive themes. We argue that if an organization uses more terms that others commonly use in the same professional community, the content from this organization would exhibit higher level coordination with others. To examine how public agencies engage in such a form of coordination during different stages of a disaster, we propose the following:

**Research Question 1:** To what extent does public agencies’ social media communication exhibit content coordination regarding COVID-19 disaster communication across different stages of the health crisis?

#### Relational-Level Coordination

The relational aspect of coordination on social media is assessed by public agencies’ retweet behaviors. As one of the most studied behaviors on social media, retweeting is the behavior of sharing or reposting another user’s original tweet to one’s own following network (Twitter, 2020). In the context of disaster agencies’ social media communication, retweeting another peer agency can be first understood as the endorsement of another agency’s disaster communication (e.g., providing disaster updates or calling for community action). In addition, the retweeting behavior enables an information exchange process, through which two agencies can achieve content-level coordination by propagating the same piece of information. During this process, the users who are retweeted can be conceptualized as “gatekeepers” or “agenda-setters” in a sense that the entire information flows within the community are shaped by the content choices of the most retweeted actors (Lee & Xu, 2018). Therefore, whether and to what extent disaster management agencies retweet one another indicates the level of relational coordination on social media. We further propose the next research question:

**Research Question 2:** To what extent does public agencies’ social media communication exhibit relational coordination through retweets across different stages of the crisis?

## Method

### Research Context

COVID-19 has been an ongoing global-scale pandemic since the first reported case in Wuhan, China, in late December 2019 (Shah et al., 2020). The first case of COVID-19 in the United States was identified on January 20, 2020, and since then, the United States has experienced multiple surges of infected cases and death rates across several states. By September 2020, the Centers for Disease Control and Prevention (CDC) reported a total of 6.41 million positive cases since March 1, 2020, and the associated disease death rate had already surpassed 200,000 (CDC, 2020). The pandemic has brought unprecedented challenges to public health and emergency management agencies at various levels. Public policies such as shelter in place and mandatory face-covering were implemented at different phases across the country to contain the disease’s rapid spread (Dave et al., 2020).

**Figure 1.**
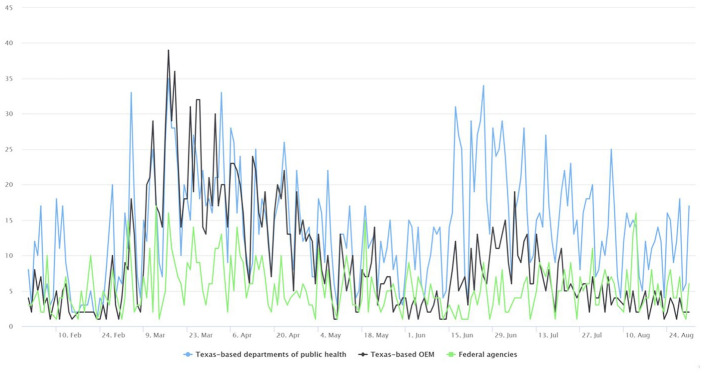
The changing volumes of COVID-19-related tweets from Texas-based departments of public health, offices of emergency management, and federal agencies.

Despite being a global pandemic, the U.S. government’s response to COVID-19 is highly localized, and the specific response significantly diverges across different jurisdictions (Dave et al., 2020). In the current study, we focus on public agencies’ COVID-19 response in Texas for the following reasons. First, Texas has been one of the disease epicenters following Governor Abbott’s state reopening measures in late April 2020 (Svitek, 2020b). The rapid escalation of the crisis prompted agencies at various levels to invest more efforts in social media communication, as evidenced by the sharp increase in the volume of tweets since the end of March 2020 (see Figure 1). Second, Texas is the second largest and second most populous state in the United States, making its emergency management and disaster response system particularly susceptible to the problem of interagency coordination. Third, Texas was one of the first states to reopen relatively early from COVID-19 shutdowns, allowing for widespread reopenings of businesses in that state on May 1, 2020 (Fernandez, 2020).

The progression of COVID-19 in Texas also followed a different trajectory than other parts of the nation. While coastal states like California, Washington, and New York were early disease hotspots, Texas did not experience surging infections until 2 months later but quickly became one of the epicenters of the outbreak in the nation (Fernandez, 2020). We therefore identified the following stages based on milestone events that were associated with major surges of new COVID-19 cases in the state: (1) *the dormant stage*, starting from Feb 3, 2020, when the Trump administration declared a public health emergency due to the coronavirus outbreak, to the end of March 2020, when the number of infected cases in Texas remained relatively low but the Governor still issued a stay-at-home order on March 24; (2) *the latent stage*, starting from April 1, 2020, to April 30, 2020; most of this phase was marked by the existing shutdown until Governor Abbott’s announcement of a first-phase reopening starting on April 27, 2020 (Svitek, 2020b); (3) *the active stage*, starting from May 1, 2020, to July 4, 2020, during which Texas experienced persistent increases in case numbers, ICU hospitalization rates, and death rates—during this phase, a mandatory face-covering advisory was issued on July 2, 2020 (Svitek, 2020a) to help curb the rapid spread; and (4) *the plateau stage*, starting from July 5, 2020, to August 31, 2020, characterized by the relative slowing down of case numbers.

### Data and Sampling

We took the following steps to collect data regarding interagency coordination on Twitter. First, we identified all active Twitter accounts of public health departments and the Office of Emergency Management (OEM) organizations at city, county, and state levels in Texas. To identify public health departments, we started with the list of health department directories through the CDC and the U.S. Department of Health & Human Services (HHS). Additionally, a list of local-level health agencies was obtained from the National Association of County and City Health Officials (2020), compiled by the organization to provide the public with COVID-19-related resources. This step identified a total of 26 Texas public health departments that actively tweeted during the studied period. We also used a list of Texas city and county names to search on Twitter and identified an additional 56 OEM organizations’ official Twitter accounts. Finally, given CDC, HHS, and National Institutes of Health (NIH) were particularly involved in the pandemic response, we further included these three federal-level agencies in the final sample, producing a total of 85 organizations.

Through the Twitter API, we used a customized Python script to collect all tweets sent by the 85 organizations between February 1, 2020, and August 31, 2020. To further extract COVID-19-related tweets, we used 79 COVID-related keywords (e.g., covid-19, coronavirus, sars-cov-2, handwashing, n95, etc.) to select relevant tweets sent by the sampled organizations from the entire study period. The final study sample consisted of 6,006 tweets (1,804 of which were retweeted content), including 3,018 by Texas-based public health departments; 1,925 from Texas OEM organizations; and 1,063 from the three federal agencies. Figure 1 below shows the volumes of tweets by each type of organization.

### Measurements

We operationalized social media–based interagency coordination in the following two ways:

#### Content-Level Coordination

To assess content-level coordination, we first used the R package textnets (Bail, 2016) to construct the shared content network of the 85 organizations, with organizational connections defined and weighted by the amount of overlap in words in their organizational tweets. In such a network, if the tweets from two organizations contained the same term (e.g., *mask*), a tie (or link) was assigned between the two organizations. A stronger tie in this network implies that the two organizations mentioned more similar topics or themes in their respective tweets. To reduce noises in the data, our text-clean process involved removing common stop words (e.g., the, a, an, to, etc.), numbers, non-English words, and generic COVID-related terms (e.g., *coronavirus*, *covid-19*, *covid*). We also focused solely on nouns, proper nouns, noun phrases, and hashtags, which were most indicative of the substantive content or discursive themes (Bail, 2016).

To measure the level of content coordination effected by each organization compared with the rest of the groups, we calculated the extent to which each organization served as a bridge in a bipartite affiliation, making connections with otherwise unconnected actors (Freeman et al., 1991). This is known as “cultural betweenness” (Bail, 2016). Organizations high in betweenness centrality in the shared content network used more common terms with the rest of the ecosystem’s organizations.

#### Relational-Level Coordination

We compiled a list of users who had been retweeted by at least one of the sampled organizations. We then used the textnets package to create a network among the 85 organizations based on the overlap of common retweeted users. Similar to the measurement of content-level coordination, betweenness centrality in this network was used as a proxy for the organization-level relational coordination, with a higher betweenness centrality score indicating that the users retweeted by the focal organization were also commonly retweeted by other organizations in the network, thereby showing a high level of relational coordination.

### Analytic Procedures

To explore how public agencies coordinated with one another on social media content during different stages of the disaster (Research Question 1), we first identified the most coordinated organizations at each stage of the pandemic. We then inductively summarized how these organizations exhibited varying levels of coordination across the four stages. To explore which types of content were commonly communicated among the selected agencies, we further presented the semantic networks based on the commonly used words and hashtags from agency tweets to contextualize the findings. The semantic networks provided a wider view of the top terms and topics that were commonly communicated by the selected public agencies in and across the stages. To answer Research Question 2, we identified the list of the most coordinated agencies in terms of retweeting common users at each disaster stage. To supplement the analysis, we discussed who the “agenda-setters” were—that is, the group of most retweeted users at each stage among the selected public agencies.

## Results

### Content-Level Coordination

Research Question 1 explored how Texas public agencies coordinated their social media content across various stages of the COVID-19 pandemic. Table 1 presents the list of agencies that exhibited the highest levels of content coordination across the four stages of the COVID-19 pandemic. At the dormant stage, when the virus was mainly affecting the West and East Coast of the United States and the local impact in Texas was limited, it was primarily local emergency management offices in densely populated metropolitan areas, such as the La Porte OEM, Galveston County OEM, Caldwell Country OEM, and Harris County OEM, that were more coordinated with the rest of the organizations in the communication ecosystem. These top OEM organizations were located near Houston, the most populated metropolitan area in the state, followed by response agencies near the Texas–Mexico border (Brownsville, Texas, and El Paso, Texas). Also noteworthy was that local public health departments were relatively less coordinated than local emergency management offices at this stage, pointing to a potential division of labor between OEM organizations and public health departments.

**Table 1.** Top 10 Agencies With the Highest Level of Content Coordination Across the Four Stages of the COVID-19 Pandemic, Indicated by Betweenness Centrality in Affiliation Network Based on Content Similarity.

Dormant stage	Latent stage	Active stage	Plateau stage
La Porte OEM	Austin PH	El Paso PH	Harris County PH
Galveston County OEM	NIH	Austin PH	Brownsville PH
Caldwell County OEM	CDC	Travis County Health & Human Services	La Port OEM
Harris County OEM	Harris County PH	NIH	Harris County OEM
Brownsville PH	Hidalgo County OEM	Montgomery County OEM	Fort Worth OEM
El Paso OEM	Denton County PH	CDC	San Antonio Metro PH
Fort Worth OEM	Texas Dept of State Health Services	Dallas OEM	Houston OEM
Hidalgo County OEM	Grayson County PH	Johnson County OEM	CDC
Plano OEM	Bastrop County OEM	Brownsville PH	Austin PH
Rusk County OEM	San Antonio Metro PH	Victoria County OEM	Travis County Health & Human Services
Wharton County OEM	La Porte OEM	Brazos County PH	Caldwell County OEM

*Note.* OEM = Office of Emergency Management.

As the outbreak spread further in the rest of the country while the state of Texas announced a phased one reopening on April 20, 2020 (the latent stage), public health departments became more coordinated than they were at the dormant stage. Specifically, national health agencies, including NIH and CDC, quickly became central organizations in the network, suggesting a greater content overlap between local agencies and the two major federal agencies, CDC and NIH. Meanwhile, other agencies that exhibited high levels of content coordination at this stage included county-level public health departments, including the Austin PHD, Harris County PHD, El Paso PHD, Travis County Department of Health & Human Services, among others. A similar pattern was observed during the active stage when the virus was raging in Texas, again highlighting a possible synergy in disaster management efforts between federal and local actors. Finally, at the plateau stage, the level of coordination between major national agencies and local health and EM agencies showed a notable decline, particularly for NIH. Local health departments and OEM organizations including Brownsville PHD, La Porte OEM, and Harris County OEM remained significant coordinators with the rest of the organizations. This pattern mirrored the dormant stage.

**Figure 2.**
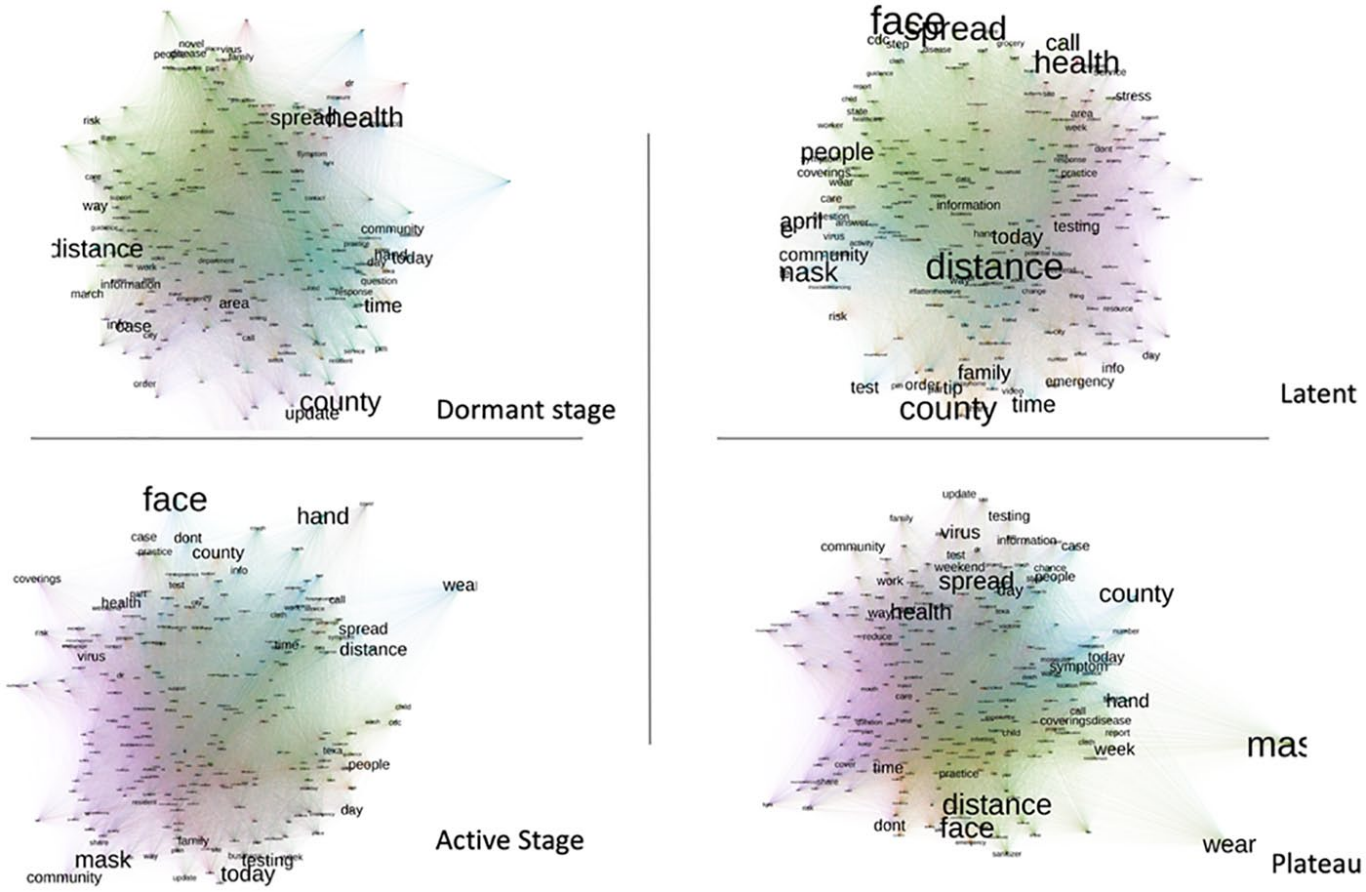
The semantic map based on the most used words from the 85 public agencies’ COVID-19 social media messages during the four pandemic stages.

A post hoc semantic analysis was conducted to compare the most coordinated content across the four pandemic stages. Figure 1 presents the respective semantic maps with the most commonly communicated terms occupying the network’s central positions. The analysis did not identify significant divergence in terms of the top terms used. Specifically, terms that indicated the communication of preventive measures—such as “face,” “mask,” “distance,” “testing,” and those about the disease risk—such as “community,” “spread,” and “symptoms” were equally present in messages at each stage (see Figure 2).

### Relational-Level Coordination

Research Question 2 examined the level of interorganizational coordination in the form of retweeting a similar group of users. Before comparing the level of such coordination across the four crisis stages, we first identified a group of users that were most frequently retweeted by the 85 organizations under study—these most retweeted users were essentially “agenda-setter” or “opinion leaders,” whereas the 85 agencies under study acted as intermediary or gatekeepers to disseminate the content to the general public.

Table 2 lists the top retweeted accounts across the four stages, and the following patterns emerged. First, across all stages, CDC and its affiliated centers, as well as state-level agencies such as the Texas Department of State Health Services, were consistently retweeted by various agencies under study, suggesting the prominent role federal agencies played as information subsidy. Second, the prominence of state-level agencies, as opposed to local operations, was notably higher at the pandemic’s latent and active stages compared with the dormant or plateau stages, indicating more prominent state-level emergency response as the pandemic became more acute. Third, although most top retweeted agencies were peer government and disaster management agencies, along with local elected officials, the Red Cross, a major nonprofit and disaster-relief organization, was frequently retweeted at the active stage of the pandemic. In contrast, news organizations were not actively engaged until the plateau stage. Four, national-level information sources, particularly the CDC, were frequently retweeted among the selected organizations at the latent stage.

**Table 2.** Top 15 Accounts Commonly Retweeted by the Selected Agencies Across the Four Stages of the COVID-19 Pandemic.

Dormant stage	Latent stage	Active stage	Plateau stage
CDC	Texas Dept of State Health Services	Texas Dept of State Health Services	CDC
Texas Dept of State Health Services	Governor Greg Abbott	CDC	Brownsville PH
Texas Economic Development	CDC	@ boemh (suspended account)	Texas Dept of State Health Services
Houston Dept of Neighborhoods	CDC Emergency Preparedness and Response	Governor Greg Abbott	Governor Greg Abbott
Harris County OEM	NIH	Mark Wiggins (lobbyists)	Surgeon General
Houston OEM	Lieutenant Governor of Texas	Texas Department of Family and Protective Service	CDC Emergency Preparedness and Response
Office of the Surgeon General	Rep. Dennis Bonnen	Seth W. Christensen (chief of communication and Texas EM)	NIH Director
Houston Mayor Sylvester Turner	Texas Military Department	Texas Secretary of State	Texas Department of Family and Protective Service
The U.S. Department of Health & Human Services	Rep. Sylvia Garcia	Red Cross	U.S. Department of Homeland Security
CDC Emergency Preparedness and Response	CDC Director Robert Redfield	Texas EM	Travis County Health & Human Services
Fort Bend County Judge	National Center for Complementary and Integrative Health	Governor Greg Abbott	Caldwell County OEM
Fort Bend County Sheriff’s Office	CDC Spanish	Victoria, Texas	Texas State Fire Marshal’s Office
Harris County Marshal’s Office	Christine Crude Blackburn (Deputy director of research program at Texas A&M)	U.S. Department of Homeland Security	The North Central Texas Emergency Communications District
Governor Greg Abbott	City of Brownsville	Surgeon General	Wes Rapaport (Texas Capitol Correspondent)
CDC Director Robert Redfield	CDC eHealth	National Weather Service, FWD	The Texas Tribune

*Note.* CDC = Centers for Disease Control and Prevention; OEM = Office of Emergency Management; NIH = National Institutes of Health.

To identify agencies that coordinated the most with the rest of the disaster communication ecosystem via retweeting, we calculated each organization’s betweenness centrality in the shared retweet user network where network ties were assigned whenever two organizations retweeted the same user. Table 3 lists the top 10 agencies at each stage of the pandemic with the highest levels of relational-level coordination. At the dormant stage, local agencies, including El Paso OEM, Bell County PHD, Kaufman County OEM, Fort Bend County PHD, and Houston City PHD coordinated with other agencies in the selected ecosystem the most. Similarly, the top relational-level coordinators at this stage are mostly located in key metropolitan areas. As the outbreak moved to the latent stage, agencies that served the greater Houston area—one of the rising epicenters in Texas—such as Harris County OEM and Harris County PHD were among the top coordinated agencies. In fact, Harris PH was the only entity to appear across all four stages in Table 3. Finally, both the active and plateau stages were characterized by a higher level of coordination between federal, state-level, and local-level agencies. For example, HHS, Texas Emergency Management, and the Texas Department of State Health Services were all among the most coordinated agencies at these two stages.

**Table 3.** Top 10 Agencies with the Highest Level of Relational Coordination Across the Four Stages of the COVID-19 Pandemic, Indicated by Betweenness Centrality in an Affiliation Network Based on Content Similarity.

Dormant stage	Latent stage	Active stage	Plateau stage
El Paso OEM	Kaufman County OEM	Harris County PH	U.S. Department of Health & Human Services
Bell County PH	Harris County PH	San Antonio Metro PH	Harris County PH
Kaufman County OEM	Harris County OEM	Fort Bend PH	Dallas County Health and Human Services
Fort Bend County PH	Denton County PH	Denton County PH	Denton County PH
Houston PH	Brownsville PH	U.S. Department of Health & Human Services	Fort Worth OEM
Brownsville PH	Houston PH	Plano OEM	Texas Dept of State Health Services
Harris County PH	Angelina County PH	Kaufman County OEM	Brownsville PH
Texas Dept of State Health Services	Tarrant County PH	Houston PH	Texas EM
Travis County Health & Human Services	Galveston County OEM	Victoria County OEM	Tarrant County PH
Angelina County PH	Texas Department of State Health Services	Angelina County PH	Ellis County OEM
Austin PH	Dallas County Health and Human Services	Texas EM	Navarro County OEM

*Note.* OEM = Office of Emergency Management.

With the two levels of coordination in mind, it is equally important to examine how coordinated a disaster response ecosystem as a whole is in its responses to the various crisis stages. We used the standard deviation of organization-level betweenness centralities as a metric to evaluate such global-scale relational coordination. If organizations at a stage had large variations in betweenness centrality scores (that is, a larger standard deviation meant that a small number of organizations were disproportionately more coordinated than other organizations), it may indicate that coordinating capacity was concentrated in selected regions or specific types of organizations.

Table 4 shows the standard deviations across the four stages for both content and relational-level coordination.^1^ As noted in Table 4, the dormant (*n* = 50) and active stage (*n* = 48) drew the highest number of participating agencies in sending original tweets concerning COVID-19. Nevertheless, in each stage, nearly more than half of the studied agencies were actively contributing content, suggesting that many agencies, likely in the less affected and populous counties, were part of the coordination network. Content-level coordination gradually increased throughout the dormant and latent stage, peaking at the active stage, and then dropped in the plateau stage. For relational-level coordination, the most active coordination occurred at the latent stage when 69 agencies retweeted at least one COVID-19-related content. The participation dropped throughout the active and plateau stage. The highest level of disparity in relational-level coordination was registered at the latent stage and decreased throughout the active and plateau stage.

**Table 4.** Ecosystem-Wide Coordination Levels Across the Four Disaster Stages, Indicated by Standard Deviation of Betweenness Centrality.

	Dormant stage	Latent stage	Active stage	Plateau stage
Content-level coordination	32.14 (*n* = 50)	39.72 (*n* = 40)	43.21 (*n* = 48)	29.96 (*n* = 42)
Relational-level coordination	58.08 (*n* = 42)	69.63 (*n* = 69)	35.23 (*n* = 29)	6.33 (*n* = 18)

## Discussion

Recognizing the vital importance of meso-level connections in citizens’ disaster communication ecology, the study explores the content- and relational-level coordination on social media among a group of Texas government and disaster management agencies during the recent coronavirus pandemic. Based on 85 public agencies’ COVID-19 tweets that span 6 months, the stage-based analysis identifies the following major findings. First, for both content- and relational-level coordination, “vertical coordination”—that is, the synchronization of social media content and retweeted information sources between federal and local agencies—appears stronger at the latent and active stages of the disaster than the dormant or plateau stage. Second, in terms of the specific themes that agencies coordinate on, the most commonly communicated themes were rather consistent throughout the four stages, focusing on preventive measures such as face-covering, testing, and social distancing. Third, state and federal-level agencies are prominent “agenda-setters” in COVID-19-related public information across almost all stages of the pandemic, as indicated by the high levels of betweenness centrality of agencies such as the CDC and the Texas Department of State Health Services in the common retweeted user affiliation networks. A related finding indicates that when it comes to top sources commonly retweeted by public health and disaster management agencies, the primary actors engaged are peer agencies rather than more diverse stakeholders such as media or private-sector organizations.

The stage-based variation regarding the level of *vertical* vis-à-vis *horizontal* coordination is worth discussing. Vertical coordination in the current study refers to the content and retweeting similarities between local, regional agencies and state, federal-level agencies. In contrast, horizontal coordination is the type that occurs between peer agencies serving the same jurisdiction levels. In terms of horizontal coordination, the results indicate that public agencies serving major metropolitan areas—such as the great Houston, Austin, San Antonio, and Dallas–Fort Worth regions, all exhibit relatively higher levels of content and relational coordination with the rest of the public organizations in the ecosystem, implying that the scope of service, as well as heightened disease risk in these areas, may be significant external forces driving such coordination.

Meanwhile, the pandemic’s urgency and acuity may be an important situational factor that encourages vertical coordination at the latent and active stages. As the outbreak spread across a greater number of Texas regions, local and regional agencies may have resorted to the upper-level agencies, such as the federal-level disaster response network, for informational and institutional support. This is especially likely given the highly uncertain and ambivalent nature of the disease. For example, the health directives regarding the coronavirus have significantly shifted over the course of the studied period (e.g., whether face-covering is necessary, the mode of disease transmission, etc.; Coleman, 2020). On the other hand, local agencies may see fewer reasons to coordinate in the dormant stage as the disease is not yet relevant. In contrast, in the plateau stage, with more knowledge and lessons learned about the disease, the need to gather information and support from higher level agencies would decline.

However, the current study identifies little stage-based variation in public agencies’ COVID-19-related tweets regarding the types of content that organizations coordinate on. The most frequently occurring words are all related to preventive and containment measures of the coronavirus outbreak, such as promoting social distancing, face-covering, and testing. This is in line with previous studies suggesting that most public health agencies’ social media communication is about “information,” such as health education, crisis updates, or broadcasting organizational programs and services (Bhattacharya et al., 2014; Neiger et al., 2013). However, more in-depth content analysis is needed to fully map the various message strategies used, which is beyond the purview of the current study.

Regarding the types of most commonly retweeted users, the current analysis identifies a group of stakeholders consisting primarily of peer government agencies. In particular, state and federal-level agencies are on the top of the most retweeted user lists, serving as “agenda-setters” during the more acute stages of the pandemic (i.e., the latent and active stages). Meanwhile, organizations from other sectors, such as nonprofits or news organizations, are not frequently engaged until the active or plateau stage. Even when the third-sector organizations enter the interorganizational information network, the relative frequency of retweeting them is still lower than that of other peer government agencies. These findings point to the following implications. First, the top-down coordination structure is still in place, especially during the more acute stages of the disaster, indicating that social media–based coordination may well still be largely driven by the on-the-ground processes (Kapucu & Garayev, 2016). Second, the findings highlight social media’s function of engaging with same-sector organizations rather than building cross-sector or media relations during public emergencies. This is consistent with Liu and Xu’s (2019) study on government agencies’ retweeting behaviors during Hurricane Harvey. The authors similarly found that government agencies prioritized peer-agency coordination over interacting with nonprofit or media organizations. The latter type of relationship building became more prominent only after the immediate threat of the disaster passed.

### Theoretical Implications for Disaster Communication Ecology

The current study makes several theoretical contributions to the disaster communication ecology framework. First, it fills a significant gap where there is only scant research attention on the meso-level connections among disaster management organizations. To theorize such meso-level connections is imperative in disaster communication because public agencies are key institutional actors that spearhead the top-down disaster management process (Kapucu et al., 2010). Identifying how well these meso-level organizational actors are connected can provide diagnostic and prognostic insights into disaster communication ecology’s performance, furthering our understanding of how individuals’ connections to such communication networks can improve disaster preparedness and coping outcomes.

Second, by taking a stage-based approach, the current study taps into the dynamic nature of communication ecology and identifies different actors that are most responsible for propagating disaster-related information at each stage. The fact that different types of actors—ranging from local, state, federal-level agencies, media organizations, to political figures—dominate the disaster communication ecology to varying degrees at each stage suggests that the communication networks are constantly evolving along with the changing environment.

Third, the four-stage construct (dormant, latent, active, and plateau stages of a crisis) offers a granularity to existing concepts of the stages of a crisis. For example, some crisis and disaster communication theorists (e.g., Spialek & Houston, 2018) have offered three-stage crisis frameworks (e.g., precrisis/crisis/postcrisis), whereas other scholars (Fearn-Banks, 2011; Madu et al., 2018) have identified five stages (e.g., detection/prevention/containment/recovery/learning). While these stage conceptualizations span the wider range of a crisis (from its infancy to its denouement), our particular construct allows for a more atomized study of crisis dynamics before the end of a crisis. Therefore, the four-stage construct offered here allows for more depth of study, especially for a crisis like COVID-19, which exemplifies a long incipient stage, followed by slow progression into following stages that feature spikes in threats (e.g., COVID cases and deaths). In other words, the four-stage construct offers a framework for a more intensive study of a “long-horizon” crisis that presents recurrences in risks and perceived threats.

### Limitations and Future Research

Like many studies, several limitations point to future research. First, while we focus on social media–based coordination at the content- and relational level, these forms of coordination do not fully capture the complex dynamics of interagency communication, which is often sustained by a multitude of activities from interpersonal meetings, phone calls, to other forms of institutionalized coordination on the ground. Recent scholarship has begun to interrogate the convergence and divergence between social media–based and off-line interorganizational communication (e.g., Liu & Shin, 2019). Therefore, future work needs to explore whether and how social media–based coordination may be related to off-line interagency coordination, such as how one type of coordination may supplement or mirror the other in the disaster communication context.

Second, in terms of understanding content-level coordination, the current study only identifies frequently occurring words without further investigating the tweets’ message type or other framing characteristics. Message features have long been a central focus in health and risk communication research (e.g., Reynolds & Seeger, 2005). Future work may help better understand the underlying mechanisms that drive content-level coordination by conducting a more in-depth thematic or framing analysis of coordinated public agency tweets.

Finally, the study does not assess the impact of interagency coordination on disaster-affected individuals or communities. After all, the theoretical framework of disaster communication ecology posits that the communication networks can equip citizens with the necessary resources to navigate the disaster (Spialek & Houston, 2018). Future work is encouraged to empirically assess the relationship between interagency coordination and citizens’ disaster-coping outcomes, such as individuals’ disaster-coping efficacy perceptions or a community’s disaster preparedness and resilience.

## Conclusion

This study finds that the unique challenge of the COVID-19 crisis results in regional Texas government and disaster management agencies synchronizing their social media content along some notable patterns. These meso-level actors are found to coordinate their Twitter feeds, both vertically and horizontally, with federal and local agencies, most markedly during the middle two stages of the four-stage concept offered here (dormant/latent/active/plateau). The content is relatively consistent across all four stages, with state and federal agencies acting as agenda setters; the presence of the media and private-sector organizations is nominal. The granularity of the four-stage flow allows scholars and practitioners to better see the fluctuations within the communication ecology pertaining to the ebb and flow of actors (e.g., local, state, federal-level agencies) that attempt to set agendas concerning the crisis narrative. As such, this work surfaces that, while broader crisis stage conceptualizations (from precrisis to postcrisis) suggest a linearity to crisis and disaster management, this study points to, and tracks, particular fluctuations in crisis management narration that is especially inherent in a novel crisis.

## Notes

1 It should be noted that not all of the 85 agencies were actively tweeting/retweeting COVID-19-related content across all stages.
